# Supplementary material for: CRISPR/cas9 Allows for the Quick Improvement of Tomato Firmness Breeding
Source: Curr Issues Mol Biol. 2024 Dec 29;47(1):9. doi: 10.3390/cimb47010009 (PMC11763693; doi:10.3390/cimb47010009)
Supplement: Supplementary file 1 [file cimb-47-00009-s001.zip › Fig.S1.pdf]

Target 1 PAM Target 2 PAM

FIS1 **Ref.** TGAGATTCTTGAAATGTTAGCTGAGG(N201)TTTCACTTGTTGATGGACATTGGATT

CR-*fis1*-OT44

1 TGAGATTCTTGAAATGTTA-CTGAGG(N201)TTTCA-TTGTTGATGGACTTTGGATT
2 TGAGATTCTTGAAATGTTA--TGAGG(N201)TTTCACTTGTTGATGGAACATTGGATT
3 TGAGATTCTTGAAATGTTA-CTGAGG(N201)TTTC-------------CATTGGATT
4 TGAGATTCTTGAAATGTTA-CTGAGG(N201)TTTCTCTTGTAGATGGAACATTGGATT
5 TGAGAATTCT-GAAAAGTTAGCTGAGG(N201)TTTCACTTGTT------AATTGGATT
6 TGAGATTCTTGAAATGTTACTG-TGAGG(N201)TTTCACTTGTAGATGGATCATTGGATT
7 TGAGATTCTTGAAATGTTACC-GAGG(N201)TTTCACTTTGTTGATGGAACTTGGATT
8 TGAGATTCTTGAAATGTTAGTCTGAGG(N201)TTTCACTTGTTGATGGAACATTGGATT
9 TGAGTTTGTTGAAATGTTT-CTGAGG(N201)TTTCACTTGTTGATGGAACATTGGATT
10 TGAGATTCTTGAAATGTTAGG-GAGG(N201)TTTCTCTTGGAGATGGAACATTGGATT
11 TGAGATT-TTGAAAAGTTAGCTTGAGG(N201)TTTC-CAAAGGGAC--AATGGATT
12 TGAGATTCTTGAAAAGTTAG-TGAGG(N201)TTTCTCTTGTAGATGGA-ATTGGATT
13 TGAGATTCTTGAAATGTTAGGATGAGG(N201)TTTCTCTTGTTGATGGACATTGGATT

CR-*fis1*-OT45

1 TGAGATTCTTGAAATGTTA-CTGAGG(N201)TTTCACTTGTTGATGG-CATTGGATT
2 TGAGATTCTTGAA--GTTAGCTGAGG(N201)TTTCACTTGTTGATGGA--TTGGATT
3 TGAGATTCTTGAAATGTTAG-TGAGG(N201)TTTCACTTGTTGATGGACATTGGATT
4 TGAGATTCTTGAAATGTT-GCTGAGG(N201)TTTCACTTGTTGATGG-ACATTGGATT
5 TGAGATTCTTGAAATGTTAGC----------------------------ATTGGATT
6 TGAGATTCTTGAAATGTTAGACTGAGG(N201)TTTCACTTGTTGATGGACATTGGATT

```
                 Target 3       PAM            Target 4        PAM
PL Ref.      GCCACTGTGGATTATTTTCGCGAGGG(N197)GGGTGGAGAACTGTTTCGGACGGTGA

CR-fis1-OT44
 1           GCCACCGT-GATTATTTTCGGGAGGG(N197)GGGTGGAGAACTCTT--GGACGGTGA
 2           GCCACTCTGGATTATTTTCGGGAGGG(N197)CGGTGGA-AACTCTTTCGGACGGTGA
 3           GCCACTGTGGATTATTTTCGCGAGGG(N197)GGGTGGAGAACTGTTTCGTGGAGAACTGTTTCGGACGGTGA
 4           GCCACTGTGGATTATTTTCGCCGAGGG(N197)GGGTGGAGAACTG-----GACGGTGA
 5           GCCACTGTGGATTATTTTCGCGAGGG(N197)GGGTGGAGAACTG------ACGGTGA
 6           GCCACTGTGGATTATTTTCG--AGGG(N197)GGGTGGAG---------GGACGGTGA
 7           GCCACTGTGGATTATTTTCGCGAGGG(N197)GGGTGGAGAACTGTTTCGGGACGGTGA
 8           GCCACTGTGGATTATTTTCG--AGGG(N197)GGGTGGAGAACTGTTT-GGACGGTGA
 9           GCCACTGTGGATTATTTTCGCGAGGG(N197)GGGTGGAGAACTGTTT---------A

CR-fis1-OT45
 1           GCCACTGTGGATTATTTTCGCCGAGGG(N197)GGGTGGAGAACTGTTT--GACGGTGA
 2           GCCACACTGTGGTTTTTTTCGCGAGGG(N197)GGGTGGAGAAAC-GTTTCGGACGGTGA
 3           GCCACTGTGGATTATTTTCGCGAGGG(N197)GGGTGG-------TTTCGGACGGTGA
 4           GCCACTGTGGATTATTTTCGCGAGGG(N197)GGGTGGAGAAC------GGACGGTGA
 5           GCCTACTGTGGATTATTTTCGCGAGGG(N197)GGGTGGAG----------GACGGTGA
 6           GCCACTGTGGATTATTTTCGCGAGGG(N197)GGGTGGAGAACTGTT--GGACGGTGA
```

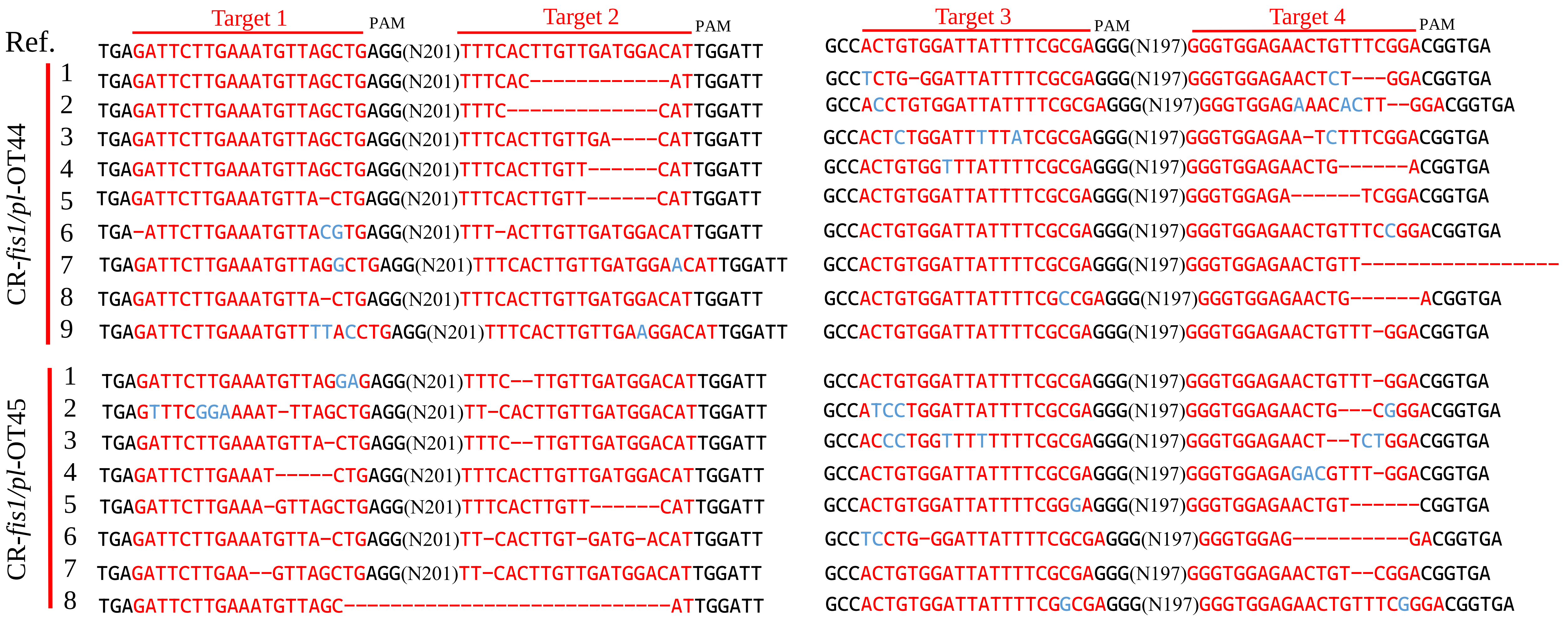
Target 1
PAM
Target 2
PAM
Target 3
PAM
Target 4
PAM
Ref. TGAGATTCTTGAAATGTTAGCTGAGG(N201)TTTCACTTGTTGATGGACATTGGATT GCCACTGTGGATTATTTTCGCGAGGG(N197)GGGTGGAGAACTGTTTCGGACGGTGA
CR-fis1/pl-OT44
1 TGAGATTCTTGAAATGTTAGCTGAGG(N201)TTTCAC------------ATTGGATT GCCTCTG-GGATTATTTTCGCGAGGG(N197)GGGTGGAGAACTCT---GGACGGTGA
2 TGAGATTCTTGAAATGTTAGCTGAGG(N201)TTTC-------------CATTGGATT GCCACCTGTGGATTATTTTCGCGAGGG(N197)GGGTGGAGAAACACTT--GGACGGTGA
3 TGAGATTCTTGAAATGTTAGCTGAGG(N201)TTTCACTTGTTGA----CATTGGATT GCCACTCTGGATTTTTATCGCGAGGG(N197)GGGTGGAGAA-TCTTTCGGACGGTGA
4 TGAGATTCTTGAAATGTTAGCTGAGG(N201)TTTCACTTGTT------CATTGGATT GCCACTGTGGTTTATTTTCGCGAGGG(N197)GGGTGGAGAACTG------ACGGTGA
5 TGAGATTCTTGAAATGTTA-CTGAGG(N201)TTTCACTTGTT------CATTGGATT GCCACTGTGGATTATTTTCGCGAGGG(N197)GGGTGGAGA------TCGGACGGTGA
6 TGA-ATTCTTGAAATGTTACGTGAGG(N201)TTT-ACTTGTTGATGGACATTGGATT GCCACTGTGGATTATTTTCGCGAGGG(N197)GGGTGGAGAACTGTTTCCGGACGGTGA
7 TGAGATTCTTGAAATGTTAGGCTGAGG(N201)TTTCACTTGTTGATGGAACATTGGATT GCCACTGTGGATTATTTTCGCGAGGG(N197)GGGTGGAGAACTGTT-----------------
8 TGAGATTCTTGAAATGTTA-CTGAGG(N201)TTTCACTTGTTGATGGACATTGGATT GCCACTGTGGATTATTTTCGCCGAGGG(N197)GGGTGGAGAACTG------ACGGTGA
9 TGAGATTCTTGAAATGTTTTACCTGAGG(N201)TTTCACTTGTTGAAGGACATTGGATT GCCACTGTGGATTATTTTCGCGAGGG(N197)GGGTGGAGAACTGTTT-GGACGGTGA
CR-fis1/pl-OT45
1 TGAGATTCTTGAAATGTTAGGAGAGG(N201)TTTC--TTGTTGATGGACATTGGATT GCCACTGTGGATTATTTTCGCGAGGG(N197)GGGTGGAGAACTGTTT-GGACGGTGA
2 TGAGTTTCGGAAAAT-TTAGCTGAGG(N201)TT-CACTTGTTGATGGACATTGGATT GCCATCCTGGATTATTTTCGCGAGGG(N197)GGGTGGAGAACTG---CGGGACGGTGA
3 TGAGATTCTTGAAATGTTA-CTGAGG(N201)TTTC--TTGTTGATGGACATTGGATT GCCACCCTGGTTTTTTTTCGCGAGGG(N197)GGGTGGAGAACT--TCTGGACGGTGA
4 TGAGATTCTTGAAAT-----CTGAGG(N201)TTTCACTTGTTGATGGACATTGGATT GCCACTGTGGATTATTTTCGCGAGGG(N197)GGGTGGAGAGACGTTT-GGACGGTGA
5 TGAGATTCTTGAAA-GTTAGCTGAGG(N201)TTTCACTTGTT------CATTGGATT GCCACTGTGGATTATTTTCGGGAGGG(N197)GGGTGGAGAACTGT------CGGTGA
6 TGAGATTCTTGAAATGTTA-CTGAGG(N201)TT-CACTTGT-GATG-ACATTGGATT GCCTCCTG-GGATTATTTTCGCGAGGG(N197)GGGTGGAG----------GACGGTGA
7 TGAGATTCTTGAA--GTTAGCTGAGG(N201)TT-CACTTGTTGATGGACATTGGATT GCCACTGTGGATTATTTTCGCGAGGG(N197)GGGTGGAGAACTGT--CGGACGGTGA
8 TGAGATTCTTGAAATGTTAGC------------------------------ATTGGATT GCCACTGTGGATTATTTTCGGCGAGGG(N197)GGGTGGAGAACTGTTTCGGGACGGTGA
