# Supplementary material for: CRISPR/cas9 Allows for the Quick Improvement of Tomato Firmness Breeding
Source: Curr Issues Mol Biol. 2024 Dec 29;47(1):9. doi: 10.3390/cimb47010009 (PMC11763693; doi:10.3390/cimb47010009)
Supplement: Supplementary file 1 [file cimb-47-00009-s001.zip › Table S1.pdf]

Table S1 The primer information for editing result detection

Primer name	Sequences (5’-3’)
Cas9-Fw	AGCGGATAACAATTTCACACAGGA
Cas9-Rv	GCAGGCATGCAAGCTTATTGG
Fw1	CAGTTGAACATCCTTTGCTAGG
Rv1	CGTGGACACTCAACGCTGAT
Fw2	TTGTTGGCGTTGTGATCCGA
Rv2	TGCTGTAGACCCCATAATCGC
